# Historical precipitation and flood damage in Japan: functional data analysis and evaluation of models

**DOI:** 10.1371/journal.pone.0318335

**Published:** 2025-02-25

**Authors:** Atsushi Wakai, Yasuaki Hijioka, Masayuki Yokozawa, Manabu Watanabe, Gen Sakurai

**Affiliations:** 1 Institute for Agro-Environmental Sciences, National Agriculture and Food Research Organization, Tsukuba, Ibaraki, Japan; 2 Center for Climate Change Adaptation, National Institute for Environmental Studies, Tsukuba, Ibaraki, Japan; 3 Faculty of Human Sciences, Waseda University, Tokorozawa, Saitama, Japan; 4 Blue and Tech Co., Ltd., Kyoto, Japan; Atlantic Technological University, IRELAND

## Abstract

The future increase of large-scale weather disasters resulting from the increased frequency of extreme weather events caused by climate change is a matter of concern. Predicting future flood damage through statistical analysis requires accurate modeling of the relationship between historical precipitation and flood damage. An analysis that considers precipitation as a time series may be appropriate for this purpose. Functional data analysis was applied to model the relationship between historical daily precipitation and daily flood damage for river basins in the Kanto and Koshin regions of Japan. Flood damage statistics from the national government and 1-km grid past precipitation data from the National Agriculture and Food Research Organization were used. The models obtained through the functional data analysis were more accurate than those derived from the simple linear regression without considering the time series of precipitation. The new models were also about four times more accurate in estimating the annual sum of flood damage, compared to the flood damage of each flood event. The accuracy of prediction was higher in recent years than in earlier years of the study period (1993–2020). The results showed that the influence of precipitation on flood damage was more apparent in recent years. This findings may imply that the progress of the river development project and the resulting improvement of the structures along the river have indirectly affected levels of flood damage associated with levels of precipitation.

## Introduction

The occurrence of extreme weather events has increased in frequency over roughly the past 20 years, and this trend can be attributed to human-induced climate change [1]. This increase has resulted in numerous hydro-meteorological disasters, including river flooding, and it has led to concerns about the potential for further increases of significant disasters in the future. In this context, modeling the relationship between historical flood damage data and meteorological data, particularly precipitation data, is crucially important to predict future flood risks. Many studies have evaluated the impact of climate change on flood risk from the perspective of flood return periods, inundation areas, and the number of people affected by the flood [2–4]. However, an insufficient number of studies have evaluated the relationship between climatic factors and the direct economic cost of flood events.

Previous studies have proposed various methods to analyze the risks of flood damage, including process-based models [5–7] and statistical models [8–11]. Process-based models have been more commonly used than statistical models for analyses of flood hazards [12–15]. Some recent studies have calculated the amount of flood damage by using the results of flood hazard analysis in a flood damage model [7,16,17]. As a part of the analysis of flood risk, the results from a flood inundation analysis have been applied to a flood damage model based on the relationship between inundation depth and extent of damage [16,17]. Several studies in Japan have quantitatively evaluated flood damage using a process-based model [18–21] that involved simulating physical quantities such as inundation depth resulting from precipitation and then calculating flood damage with reference to the Manual for Economic Evaluation of Flood Control Investment (draft) provided by the Japanese government.

In contrast, the statistical models to estimate flood damage directly from precipitation have been proposed because estimates of flood damage can be derived from relevant statistics and insurance payments. For instance, Cortès et al. [9] modeled the relationship between historical precipitation and the probability of flood damage estimated from data on insurance claims, and Davenport et al. [10] directly modeled the relationship between historical precipitation and flood damage. Bhattarai et al. [11] developed a model to estimate annual damage by modeling the probability of occurrence of damage and the damage cost using data such as historical precipitation and flood damage.

Another advantage of using a statistical model is that the researchers can model the relationship between the actual damages recorded in the past statistical data and climatic factors. For example, most previous studies that have not used statistical models have calculated only the amount of flood damage to common assets such as housing, household commodities and business assets; there have been considerably fewer cases in which the amount of damage to public engineering facilities has been calculated. In contrast, statistical records have usually provided a more detailed categorization of the damage, and that information has enabled the relationship between climatic factors and more specific kinds of flood damage to be modeled.

Previous studies using statistical methods have focused on maximum or average precipitation over specific time periods (e.g., hourly [9], daily [11], or monthly [10]). However, precipitation is inherently a time series, and it is also important to consider the effect of the time series of precipitation leading up to flood events; a suitable method for analyzing a time series of precipitation should therefore be applied.

To address this issue, functional data analysis [22], a statistical method that can be used to address the relationships between functionalized data, was used in this study. The explanatory and response variables were treated as functions, not scalar or vector values, in this analysis. For instance, daily precipitation functions can be obtained by functionalizing discrete daily precipitation data for a certain period. Use of this method enabled treatment of the precipitation data as a time series function rather than using summary statistics of the time series.

The objective of this study was to model the relationship between a time series of precipitation and the amount of flood damage in river basins. To achieve this goal, functional data analysis was applied to model the relationship between historical daily precipitation and flood damage to public engineering facilities for each flood event for each river basin in the Kanto and Koshin regions of Japan. The accuracy of the obtained models was then evaluated and the differences in accuracy were compared for different temporal divisions of the study period.

## Materials

### River basin data

Data with 100-m resolution for river basins in the Kanto and Koshin regions of Japan were obtained from the Digital National Land Information provided by the Ministry of Land, Infrastructure, Transportation and Tourism (MLIT) [23]. The data consisted of items such as the code and name of each river system (i.e., a network of a main river, tributaries and distributaries) and each river as well as the polygon geometry within each river basin. The data were used to create precipitation data, slope data and population data in each river basin.

### Precipitation data

Historical daily precipitation data with 1-km resolution were obtained from the Agro-Meteorological Grid Square Database provided by the National Agriculture and Food Research Organization [24]. The data consisted of the 1-km grid code for each parcel of land and daily precipitation for each year from 1993 to 2020. For each river basin, the average daily precipitation was calculated by simply averaging the daily precipitation of the grid points included in each river basin. The spatial and temporal resolution was one river and one day, respectively, because the historical data on flood damage was organized by each date and each river for each flood event.

### Flood damage data

Historical flood damage data for public civil facilities were obtained from the Statistics of Flood Damage provided by the Water and Disaster Management Bureau of the MLIT [25]. The data consisted of the cost of damage to public civil facilities and included items such as river structures, sabo facilities, and bridges for each flood event and each river from 1993 to 2020. Sabo facilities store sediment coming from upstream and/or prevent sediment deposited on the riverbed from flowing out. The data also included information such as the dates of flood events and the codes and names of river systems and rivers. In this study, the cost of damage to all the affected facilities were summed for each flood event and each river; the costs were adjusted to the value of the Japanese yen in 2020.

### Slope data

Observed slope data with 1-km resolution were obtained from the Digital National Land Information provided by MLIT [26]. The data consisted of items such as mean slope angles, mean elevations, and polygon geometries. The mean slope rate for each river basin was calculated by averaging the slope rate obtained based on the slope angles of the grid points in each river basin.

### Population data

Population data at the municipal level were obtained from the database provided by the Statistics Bureau of the Ministry of Internal Affairs and Communications [27]. The data contained information such as the population of each municipality in each prefecture. The population in each river basin was calculated by summing the populations of all municipalities within the river basin.

### Target region

**Fig 1:**
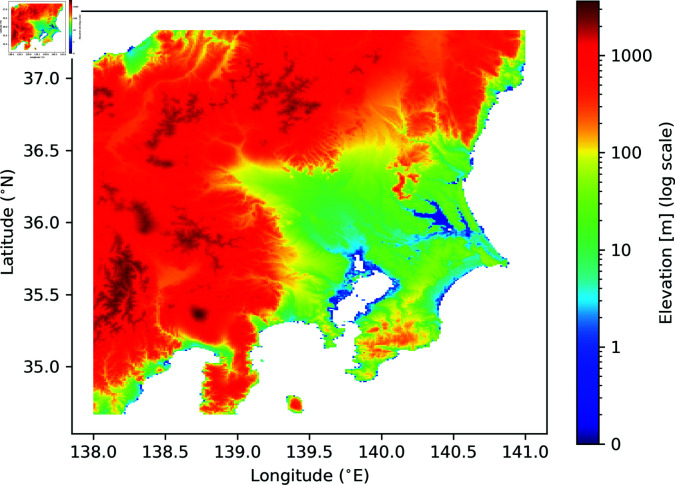
Elevation map of the Kanto and Koshin regions of Japan. The cooler colors indicate lower elevations, and the warmer colors indicate higher elevations. Created by the author based on the elevation 1-km grid data published by the national government [26]. The copyright holder’s requirements are compatible with a CC BY 4.0 license [28].

The study focused on the Kanto and Koshin regions of eastern Japan (Fig 1). These regions include the prefectures of Tokyo, Ibaraki, Tochigi, Gunma, Saitama, Chiba, Kanagawa, Yamanashi, and Nagano. The regions contain the largest plain in Japan and include 178 river systems and 2,998 rivers. This study covered seven river systems under the control of the national government and three groups of river systems under the control of each local government (Ibaraki, Chiba, and Kanagawa prefectures).

## Methods

### Modeling

The objective of the study was to model the relationship between a time series of daily precipitation in each river basin and the amount of flood damage for each date and for each river basin. Functional linear regression analysis [22], which can functionalize discrete time series data, was used to treat raw time series data.

The time series of the accumulation of mean daily precipitation for a certain number of days just before each flood event was used as the explanatory variable; the total cost of the repair of the public civil engineering facilities damaged by each flood event was used as the response variable. Precipitation as the explanatory variable was quantified based on the mean daily precipitation in each river basin as follows: first, a time series of mean daily precipitation was extracted for a specific number of days before each flood event; then, the extracted time series of mean daily precipitation was accumulated. Flood-control projects based on plans for river improvement have been implemented by both national and local governments for many years [29,30]. It was not appropriate to apply a single statistical model to all target periods, even though flood improvement projects had been ongoing for decades. The target period should be therefore divided into at least two parts, with separate models for each part. The division of the study period into three or more parts resulted in an insufficient number of flood events for each period because the requisite number of data points was not attained. The decision was therefore made to divide the study period into two parts. Several division points were considered to determine the optimal one, and then the difference between the two periods was examined based on the accuracy of each model obtained from functional data analysis. The following equation described the assumed model:


log ⁡ 10(y(i,b),r,p∕pop(b))=αr,p+ ∫ -m0βr,p(t)v(i,b),r,p(t)dt+ϵ(i,b),r,p (1)

Here, $y_{(i,b),r,p}∕pop_{\left(b\right)}$ represents the * log* ⁡  -transformed total cost of damage per capita for flood event *i* of river basin *b* during the first part  ( *p* = 1 )  or the second part  ( *p* = 2 )  of the target period (1993–2020); *r* represents the river system that includes river basin *b*  ( *r* = 1 , … , 10 ) ; the constant $\alpha_{r,p}$ is the intercept in the equation; $\beta_{r,p}(t)$ represents the regression coefficient function constructed by B-spline basis expansions [31]; and $v_{(i,b),r,p}(t)$ represents functions constructed by B-spline basis expansions [31] for Manning’s flow velocity vector, $v_{(i,b),r,p}$, obtained from the following equation:


$$\begin{array}{c} v_{(i,b),r,p}=\frac{1}{n}{\left(x_{(i,b),r,p}\right)}^{\frac{2}{3}}I_{b,r}^{\frac{1}{2}} \end{array}$$ (2)

Here, *n* is Manning’s roughness coefficient, $x_{(i,b),r,p}$ is a vector for the time series of the accumulation of daily precipitation up to *m* days before the flood event; *m* is the number of days considered in the analysis; $I_{b,r}$ is the mean slope in the river basin; and $\epsilon_{(i,b),r,p}$ is the distribution of errors, which was assumed to be normal. Precipitation was converted to the Manning’s flow velocity based on the slope of the river basin to take account of topographical differences between the upstream and downstream reaches of each river basin.

In the functional data analysis, we functionalized the time series of flow velocity transformed from accumulated daily precipitation using Manning’s equation. Because the time series was accumulated values, the values increased monotonically. For this kind of data, applying a B-spline is preferable as the basis function rather than a Fourier series function that is preferable if the time series is periodic and stationary. The order and number of basis functions were used as the parameters for the B-splines. The order was determined to be four. The optimal number of basis functions was identified through cross-validation. The number of knots was automatically identified based on the order and the number of basis functions. The knots were spaced at equal intervals.

The log-transformed total cost of damage per unit area and per gross regional product (GRP) were also considered as response variables in the modeling process, in addition to the log-transformed total cost of damage per capita. The damage per unit area was less than that per capita, and the damage per GRP was almost equal to that per capita, in terms of the accuracy of cost of damage. The damage per capita is therefore presented here.

To summarize, $\beta_{r,p}(t)$ was estimated for each river system  ( *r* = 1 , … , 10 )  and for each period  ( *p* = 1 , 2 ) . The $\beta_{r,p}(t)$ was modeled as follows:


$$\begin{array}{c} \beta_{r,p}(t)=\overset{J}{\underset{j=1}{\sum }}w_{r,p,j}\phi_{j}(t) \end{array}$$ (3)

Here, the $w_{r,p,j}$ are parameters of the basis function to be determined, and $\phi_{j}(t)$ is a B-spline basis function [31]; *J* is the number of basis functions, and the number of flood events is fixed for each river system and for each period.

Seventeen different divisions of the study period were assessed: 1993–1998 and 1999–2020, 1993–1999 and 2000–2020, ..., and 1993–2014 and 2015–2020. In addition, the analysis for the number of days before each flood event (*m* in Eq (1)) was varied between 5 and 14 days. The optimal division into periods was determined by evaluating the model for each division into periods for all values of m. The optimal value of m was then determined by evaluating the model with the optimal division into periods for each value of m.

To compare the precision of this method with that of another method, classical simple regression analysis was also performed following the same procedure. Unlike functional regression analysis, total precipitation up to a certain number of days before each flood event was used as the explanatory variable.

### Evaluation of the model

To evaluate the model obtained using functional linear regression analysis, the coefficient of determination for observed and predicted flood damage was calculated as follows:


R2=1-∑in{log ⁡ 10(y(i,b),r,p∕pop(b))- log ⁡ 10(ŷ(i,b),r,p∕pop(b))}2 ∑in{log ⁡ 10(y(i,b),r,p∕pop(b))- log ⁡ 10(ȳ(i,b),r,p∕pop(b))}2 (4)

Here, $y_{(i,b),r,p}$ is the observed flood damage, ${ŷ}_{(i,b),r,p}$ is the predicted flood damage, ${ȳ}_{(i,b),r,p}$ is the average of the observed flood damage, $pop_{\left(b\right)}$ is the population in each river basin and n is the number of flood events.

The division into periods that resulted in the highest *R*^2^ was selected for each of the number of days before each flood event (*m* in Eq (1)). For the selected optimal division into periods, the number of days (*m*) that resulted in the highest *R*^2^ value was then selected from a range of values between *m* = 5 and 14. This procedure was conducted for each river system. The value of *m* and the type of the division of the entire period (1993-2020) therefore differed among the river systems.

Next, to compare the results from the functional linear regression analysis with those from the simple regression analysis, the value of *R*^2^ covering all the river systems was calculated for each division into periods for each method. In this case, the *R*^2^ values from the functional regression analysis were calculated based on the models corresponding to a time series of precipitation for the optimal number of days before each flood event.

The total cost of the damage to each river system in a year was then calculated to evaluate the accuracy of the annual sum of flood damage. In this analysis, the identity link function was used instead of the log link function. The optimal value of *m* and the division into periods were estimated using the *R*^2^ of the observed and estimated total annual flood damage.

## Results

### Accuracy of the model and selected division into periods for each river system

As described in the Methods section, the best combination of the division into periods and the number of days before each flood event (*m* in Eq (1)) was first identified. The combination that led to the maximum *R*^2^ was 1993–1999 for the first period, 2000–2020 for the second period, and 14 days for the number of days (*m* = 14). Table 1 shows the *R*^2^ value for each combination of the division into periods and the number of days before each flood event.

**Table 1:** The *R*^2^ values obtained through the functional regression analysis.

Division into periods			Number of days before each flood event									
**ID**	**First part**	**Second part**	**5**	**6**	**7**	**8**	**9**	**10**	**11**	**12**	**13**	**14**
1	1993–1998	1999–2020	0.205	0.202	0.220	0.223	0.222	0.229	0.231	0.236	0.236	0.240
2	1993–1999	2000–2020	0.219	0.216	0.227	0.231	0.229	0.238	0.243	0.244	0.248	**0.252**
3	1993–2000	2001–2020	0.218	0.215	0.226	0.226	0.223	0.233	0.237	0.242	0.247	0.250
4	1993–2001	2002–2020	0.210	0.209	0.222	0.223	0.221	0.229	0.240	0.244	0.244	0.250
5	1993–2002	2003–2020	0.207	0.206	0.219	0.218	0.217	0.225	0.240	0.243	0.243	0.248
6	1993–2003	2004–2020	0.204	0.205	0.217	0.216	0.214	0.221	0.237	0.240	0.240	0.249
7	1993–2004	2005–2020	0.199	0.202	0.212	0.214	0.214	0.217	0.232	0.234	0.234	0.236
8	1993–2005	2006–2020	0.194	0.198	0.210	0.211	0.212	0.215	0.229	0.232	0.232	0.234
9	1993–2006	2007–2020	0.193	0.198	0.208	0.209	0.210	0.212	0.227	0.229	0.229	0.231
10	1993–2007	2008–2020	0.188	0.192	0.204	0.204	0.204	0.208	0.231	0.229	0.225	0.228
11	1993–2008	2009–2020	0.188	0.189	0.203	0.203	0.203	0.207	0.230	0.229	0.225	0.225
12	1993–2009	2010–2020	0.185	0.187	0.200	0.200	0.201	0.205	0.227	0.225	0.222	0.223
13	1993–2010	2011–2020	0.186	0.188	0.201	0.201	0.201	0.206	0.226	0.224	0.221	0.222
14	1993–2011	2012–2020	0.182	0.186	0.192	0.194	0.196	0.199	0.217	0.217	0.212	0.213
15	1993–2012	2013–2020	0.182	0.187	0.193	0.195	0.195	0.200	0.216	0.216	0.214	0.217
16	1993–2013	2014–2020	0.179	0.185	0.191	0.193	0.193	0.197	0.210	0.212	0.210	0.211
17	1993–2014	2015–2020	0.179	0.185	0.191	0.193	0.193	0.197	0.210	0.212	0.210	0.211

The *R*^2^ value for each division into periods for each value of the number of days before each flood event. The *R*^2^ values were calculated for the entire study period (1993–2020) for all the river systems. ID denotes a pair of two periods for each division into periods. The bold text highlights the optimal division into periods and number of days.

**Fig 2:**
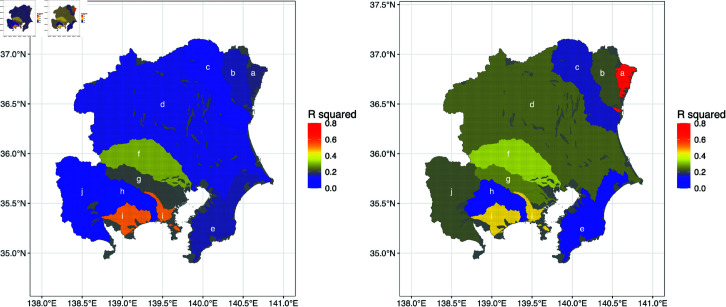
Distribution maps of the coefficient of determination, *R*^2^. The *R*^2^ values were for each river system for the optimal division into periods and the optimal number of days before each flood event. The left and right panels show the periods 1993–1999 and 2000–2020, respectively. The cooler colors show the lower *R*^2^ and the warmer colors show the higher *R*^2^. Letters represent river systems, as listed in Table 2. Processed and created by the author based on the river basin grid data published from the national government [23]. Republished from [23] under a CC BY license, with permission from National Spatial Planning and Regional Policy Bureau of MLIT, original copyright 2009.

Distribution maps of *R*^2^ for each river system were plotted for the optimal division into periods (1993–1999 and 2000–2020) corresponding to the daily precipitation time series for the 14 days before each flood event (Fig 2). The *R*^2^ values of the second part of the period (2000–2020) were generally higher than those of the first part (1993–1999). Also, the *R*^2^ values differed among river systems.

Table 3 listed the optimal division into periods and the coefficients of determination for both parts of each period for each river system. The optimal division into periods differed for some river systems, and each river system exhibited different *R*^2^ values between the first and the second parts of the study period. Also, the *R*^2^ values differed among river systems. (For reference, the cases of the flood damage per unit GRP and per unit area on a response variable are included in the Supporting Information (S1 Table and S2 Table).)

#### The curve of the regression coefficient function.

**Fig 3:**
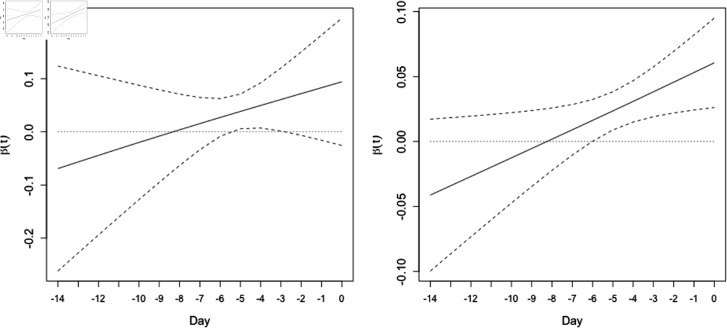
An example of a simple regression coefficient function. The function *β* ( *t* ) , with 95*%* confidence intervals for the Tama River water system (830305) for the optimal division into periods and the optimal number of days before each flood event. The left and right panels show results for the periods 1993–1999 and 2000–2020, respectively. The horizontal and vertical axes represent the number of days before each flood event and the values of *β* ( *t* ) , respectively. Day 0 represents the day when each flood event occurred. Solid lines represent the average of the regression coefficient function; dashed lines denote 95*%* confidence intervals. This indicates a simple curve shape.

**Fig 4:**
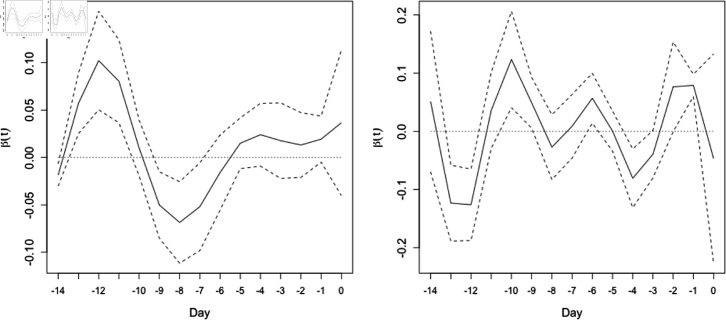
An example of a complex regression coefficient function. The function *β* ( *t* ) , with 95*%* confidence intervals for the Fuji River water system (830308) for the optimal division into periods and the optimal number of days before each flood event. The left and right panels show results for the periods 1993–1999 and 2000–2020, respectively. The horizontal and vertical axes represent the number of days before each flood event and the values of *β* ( *t* ) , respectively. Day 0 represents the day when each flood event occurred. Solid lines represent the average of the regression coefficient function; dashed lines denote 95*%* confidence intervals. This indicates a complex curve shape.

Figs 3 and 4 show examples of the curves of the regression coefficient function for two river systems. The coefficients were estimated performing functional regression analysis. The curves in Figs 3 and 4 exhibited simple and complex curve shapes, respectively. These simple and complex curve shapes corresponded to the simplicity and complexity of the relationship between the time series of precipitation and the amount of flood damage, respectively. The Supporting Information (S1 Fig–S8 Fig) shows curves of the regression coefficient function for other river systems.

**Table 2:** Codes and names of river systems

River system		
Letter	Code	Name
a	080000	Ibaraki Pref.
b	830301	Kuji River
c	830302	Nakagawa River
d	830303	Tone River
e	120000	Chiba Pref.
f	830304	Arakawa River
g	830305	Tama River
h	830307	Sagami River
i	140000	Kanagawa Pref.
j	830308	Fuji River

The information is corresponding to the letters in Fig 2. The codes beginning with "83" denote the river systems under the control of the national government. The others denote the river system groups under the control of the local governments. Abbreviation: Pref., Prefecture.

### Comparison of functional linear regression with simple linear regression

**Fig 5:**
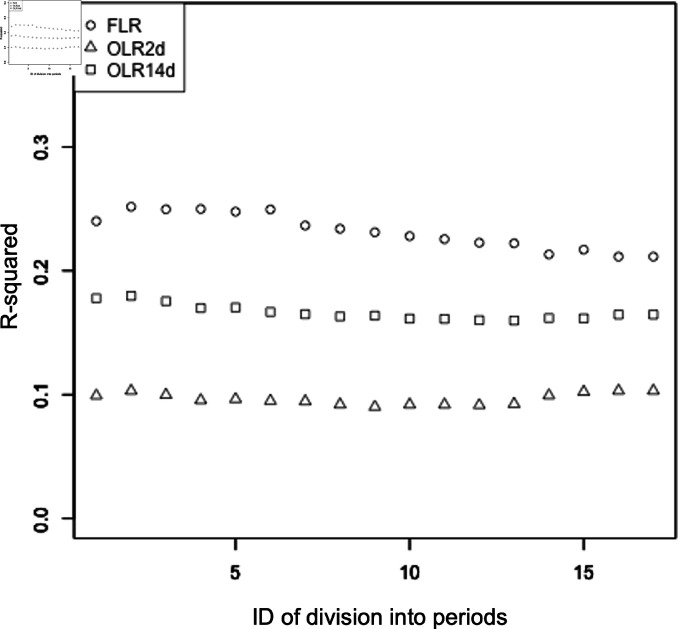
Comparison of the *R*^2^ values for each method for each division into periods. The horizontal and vertical axes show the ID of division into periods and the corresponding values of *R*^2^, respectively. Circles labeled as FLR represent the functional regression analysis; triangles labeled as OLR2d and squares labeled as OLR14d represent the simple regression analysis where the explanatory variable was the total precipitation for 2 and 14 days before each flood event, respectively. Division into periods are numbered as in Table 1.

The *R*^2^ values of the functional regression analysis were compared with those of the classical simple regression analysis for all divisions into periods ( Fig 5). For the functional regression analysis, the explanatory variable was the precipitation function for the 14 days before each flood event; for the classical simple regression analysis, the explanatory variable was the total precipitation for 2 or 14 days before each flood event. The *R*^2^ values of the functional regression analysis were higher than the values for the other two analyses for all divisions into periods.

### Prediction of annual sum of flood damage

**Fig 6:**
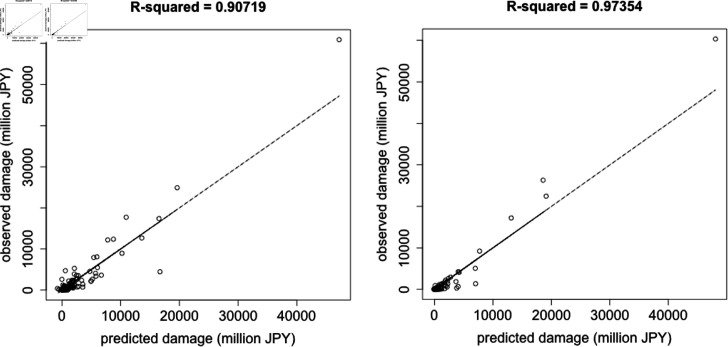
Correlation between the annual sum of observed and predicted flood damage. The plots are for the optimal division into periods and the optimal number of days. The results encompass all river systems. The left and right panels show the periods 1993–2008 and 2009–2020, respectively. The horizontal and vertical axes represent the predicted and observed flood damage, respectively. The *R*^2^ values were calculated for each of the two periods. Abbreviation: JPY, Japanese yen; 2020 being used as the basis.

The annual sum of flood damage for each river system was calculated; Fig 6 shows the correlation between observed and predicted values. (S3 Table) in Supporting Information lists the *R*^2^ values for all the river systems calculated by varying the combination of division into periods and the number of days before each flood event. The *R*^2^ values in S3 Table were calculated for the entire study period (1993–2020). The optimal division into periods was 1993–2008 and 2009–2020. The number of days before each flood event with the highest *R*^2^ for this optimal division into periods was 11 days. The *R*^2^ values in Fig 6 were calculated for each of the two periods for all the river systems. The fact that both parts of the period exhibited *R*^2^ values greater than 0.9 indicated a high level of accuracy. Furthermore, the *R*^2^ value of the second part of the period was larger than that of the first part, in the same way as the case of flood damage for each flood event, as illustrated in Fig 2.

## Discussion and conclusion

In this study, functional regression analysis was applied to model the relationship between time series of precipitation and flood damage to public engineering facilities. The estimated models were evaluated using the coefficient of determination. The applicability of using the functional data analysis was evaluated by comparing *R*^2^ values of the models generated by it and by another method. The applicability of the model was also evaluated by comparing the annual sum of flood damage between estimated values and observed values.

Data-driven methods can be classified into two principal categories: statistical and machine-learning methods. Statistical models include models such as classical regression models and generalized additive models [32]. Machine learning models include, for example, decision trees [33] and neural networks [34]. Functional data analysis falls in the category of statistical models. Many statistical methods have a clear functional form within the model and can evaluate the degree of influence of the explanatory variables on the response variable. However, it is challenging to model complex relationships, and in principle, time series data cannot be used as variables other than for functional data analysis. In contrast, machine learning methods can model complex relationships and are expected to be highly accurate. Machine learning methods have been applied to numerous flood risk studies in recent years, primarily studies of flood hazard analyses, including the prediction of flood discharge, inundation depth, and so forth [35–37]. However, it is difficult to grasp the structure of the internal functional form of the model.

This study revealed the extent to which a precipitation time series affects flood damage. Precipitation data are essentially time series data. The accuracy of the model may be enhanced by using the time-series data themselves rather than representative values such as maximum and average values. Functional data analysis was selected as the method to achieve this. By using this method, we were able to grasp the relationship between precipitation time series and flood risk. This study also suggested that the functional data analysis has high estimation accuracy relative to a statistical method such as simple regression. High estimation accuracy may be achieved using machine learning methods, but it is difficult to clearly understand the relationship between precipitation and flood risk. By using functional data analysis, we can grasp the relationship between the precipitation time series and the risk of flooding and thereby achieve relatively high estimation accuracy.

To examine the model’s accuracy using functional regression analysis, the *R*^2^ values of the obtained model were compared with those of the model using classical simple regression analysis (Fig 5). The functional regression analysis provided more accurate results for prediction than simple regression analyses. Although the simple linear regression treated the total precipitation during a certain time period, the current analysis treated the precipitation time series itself. The better accuracy may be attributed to this difference. The results suggest that consideration of the time series of precipitation is effective to some extent for statistical modeling of flood damage. Previous studies using statistical analyses treated monthly total precipitation and maximum total precipitation over five consecutive days within each month [10], maximum 24-hour precipitation [9], and daily precipitation exceeding medium-scale rainfall intensity [11]. Those studies did not treat precipitation time series itself.

Functional linear regression analysis was applied to estimate regression coefficient functions (*β* ( *t* ) ), as shown in Figs 3 and 4. The regression coefficient function reflects how much a time series of an explanatory variable affects a response variable; the results thus suggest that precipitation on days closer to a flood event has a greater effect on flood damage (Fig 3). This result is intuitively correct and suggests that analysis of precipitation time series can be meaningful. In contrast, Fig 4 suggests that precipitation on days close to a flood event and days farther away from the flood event have a large effect on flood damage. This result is challenging to interpret. Further study will be necessary to understand the implications of the regression coefficient functions.

The *R*^2^ values of the first and second parts of the study period were compared for each river system (Fig 2). The result showed that the model was generally more accurate for the second part than for the first part for these river systems. In Japan, the river improvement policy has made a major change because of the revision of the River Law in 1997. Since then, the national and local governments have made new river improvement plans, and they have continuously implemented numerous flood control projects [29,30] (S9 Fig). This fact seems to have led to an annual increase in the number of river structures constructed for flood control. The implication is that the number of river structures exposed to each flood event may be greater in the current period than in the past period. It may suggest that there is a clearer relationship between precipitation and the cost of flood damage. Although the exact reason for the greater accuracy during the second period should be analyzed in future studies, the improved accuracy in the second part of the study period may be related to the progress made in flood control projects.

In addition, the optimal division into periods and the *R*^2^ values were compared for each river system and for the optimal number of days before each flood event (Table 3). The optimal division of the study period and the accuracy differed among river systems. These differences may have arisen because the budgets for and progress in flood control projects differed between urban and rural areas. The implication may be that the effects of river improvement were observed at different times. It appears that a larger budget has been allocated to the river systems such as the Arakawa River (830304) and Tama River (830305), which include metropolitan areas (S10 Fig). However, there is a limitation to identification of the cause because relevant information covers only the last several years of the study period. The optimal division into periods and the accuracy of the models may be somehow related to the allocation of the flood control project budget for each river system.

Previous studies using statistical analysis have addressed precipitation and flood damage data with longer periods of approximately 20 years [9,11] or 30 years [10]. However, those studies have not considered change over the years in their models. Adopting the same model over the long term may not be appropriate. As mentioned in the previous two paragraphs, this study may be more reasonable because the results showed that the division of the study period into two parts revealed a difference in the accuracy of the model for each part of the study period.

This study found that using functional data analysis improved the accuracy of the prediction of flood damage compared to classical analytical methods. However, the accuracy itself was still relatively low (Fig 5). There may be some factors that explain this result. The first reason may be that precipitation was used as an explanatory variable instead of river discharge. In principle, river discharge can directly affect the degree of damage level of river structures. Using river discharge as an explanatory variable for detailed analysis is thus more suitable. However, this study used precipitation as an explanatory variable for simplicity in order to model the whole area comprehensively. To compute river discharge, some studies have precisely modeled river structures to perform hydrological and hydraulic simulations [21,38,39]. In contrast, this study could estimate flood damage more simply by using precipitation. This ability may be a significant advantage of the methodology. The second reason may be the assumption of the same relationship between precipitation and flood damage in each river system. While each river may have a different relationship between these variables, this analysis assumed that the relationships were the same for the rivers in each river system. In addition, this study processed river basin data by identifying rivers under the control of national and local governments based on river codes defined by the MLIT. However, some rivers are actually divided and managed by some organizations, such as national and local governments, especially for significant rivers. The status of river improvement may therefore vary as a function of the section for which each organization is responsible. That may be one of the reasons why statistical variations are large in flood damage corresponding to precipitation. Ideally, each river should be modeled instead of each river system. However, this approach can result in a lack of data for flood damage required in the analysis. In this study, the data from each river were pooled and organized by river system prior to analysis.

To evaluate the accuracy of the obtained models with coarser temporal resolution (annual) than each flood event described above, the correlation between the annual sum of observed and predicted flood damage was also examined for all the river systems (Fig 6). Although some predicted values were more than ten times the observed values or less than one-tenth of the observed values, overall, highly accurate estimations of the annual sum of flood damage were obtained. This may reflect the fact that summing up the flood damage from each year resulted in smoothing out of the variation in flood damage predicted by the models.

**Table 3:** Optimal division of study period and *R*^2^ values for each period and for each river system.

River system		Division into periods		*R* ^2^	
Code	Name	First part	Second part	First part	Second part
080000	Ibaraki Pref.	1993–1998	1999–2020	0.302	0.491
120000	Chiba Pref.	1993–2002	2003–2020	0.139	0.118
140000	Kanagawa Pref.	1993–1998	1999–2020	0.539	0.450
830301	Kuji River	1993–1999	2000–2020	0.151	0.220
830302	Nakagawa River	1993–1998	1999–2020	0.178	0.120
830303	Tone River	1993–2001	2002–2020	0.122	0.269
830304	Arakawa River	1993–2000	2001–2020	0.294	0.341
830305	Tama River	1993–2003	2004–2020	0.386	0.351
830307	Sagami River	1993–2010	2011–2020	0.150	0.110
830308	Fuji River	1993–1999	2000–2020	0.100	0.218

The case that a response variable was flood damage per capita in each river basin. Abbreviation: Pref., Prefecture.

A functional data analysis approach may be scalable to other regions, countries or to larger datasets, provided that the datasets are compatible with the items, quantity, and resolution of the data used in this study. At least, the approach can be applied to other regions in Japan. However, as the size of datasets increases, the computational costs also increase. When the required computation time is not feasible, it may be necessary to consider using high-performance computing. Furthermore, it is currently challenging to determine the extent to which the findings from this study can be generalized to other regions or countries with different hydrological and meteorological conditions. Therefore, the validation of the findings needs to be considered in future research.

Functional data analysis was applied to model the relationship between precipitation time series and flood damage. The method produced the a more accurate model than the model obtained via a classical method. The accuracy of the estimated models was good for the annual sum of flood damage. Also, the method showed in detail the effect that precipitation time series can have on flood damage from the curves of the regression coefficient function (*β* ( *t* ) ) estimated in the analysis. It effectively divided the study period based on the different accuracies of the estimated models and considered the changes that occurred over the years. The difference may reflected progress and budgets for river improvement projects. The conclusion is that functional data analysis can produce statistical models with better accuracy for flood risk analysis. A more detailed examination of the regression coefficient functions is however necessary because their current interpretation remains incomplete. Additionally, it is essential to validate the applicability of functional data analyses to other regions in Japan.

To predict flood damage in the future, it will be essential to predict a flood event. Such a prediction can be achieved through the evaluation of the probability of flood events using a method such as logistic regression analysis. Knowledge of the probability of a flood event will enable estimation of the flood damage caused by the flood event directly.

## Supporting information

S1 Table Optimal division of study period and *R* ^2^ values for each period and for each river system. The example that a response variable was the damage per gross regional product (GRP) in each river basin. Abbreviation: Pref., Prefecture. The historical GRP data at the prefectural level were obtained from a database provided by the Cabinet Office (Prefectural economic statistics; 2024. https://www.esri.cao.go.jp/jp/sna/sonota/kenmin/kenmin_top.html). The data contain information such as the gross product for each prefecture in each year. The GRP in each river basin was calculated by multiplying the GRP for the target region by the ratio of the population within the river basin to the population for the target region. The GRP was adjusted to the value of the Japanese yen in the year 2020.
(TIF)

S2 Table Optimal division of study period and *R* ^2^ values for each period, for each river system. The example that a response variable was the damage per unit area in each river basin. Abbreviation: Pref., Prefecture.
(TIF)

S1 Fig The regression coefficient function for the water system 080000. The function *β* ( *t* ) , with 95*%* confidence intervals for the Ibaraki Prefecture water system (080000) for the optimal division into periods and the optimal number of days before each flood event. The left and right panels show 1993–1999 and 2000–2020, respectively. The horizontal and vertical axes represent the number of days before each flood event and the values of *β* ( *t* ) , respectively. Day 0 represents the day when each flood event occurred. Solid lines represent the average regression coefficient function; dashed lines denote 95*%* confidence intervals.
(TIF)

S2 Fig The regression coefficient function for the water system 120000. The function *β* ( *t* ) , with 95*%* confidence intervals for the Chiba Prefecture water system (120000). The description is the same as for S1 Fig.
(TIF)

S3 Fig The regression coefficient function for the water system 140000. The function *β* ( *t* ) , with 95*%* confidence intervals for the Kanagawa Prefecture water system (140000). The description is the same as for S1 Fig.
(TIF)

S4 Fig The regression coefficient function for the water system 830301. The function *β* ( *t* ) , with 95*%* confidence intervals for the Kuji River water system (830301). The description is the same as for S1 Fig.
(TIF)

S5 Fig The regression coefficient function for the water system 830302. The function *β* ( *t* ) , with 95*%* confidence intervals for the Naka River water system (830302). The description is the same as for S1 Fig.
(TIF)

S6 Fig The regression coefficient function for the water system 830303. The function *β* ( *t* ) , with 95*%* confidence intervals for the Tone River water system (830303). The description is the same as for S1 Fig.
(TIF)

S7 Fig The regression coefficient function for the water system 830304. The function *β* ( *t* ) , with 95*%* confidence intervals for the Arakawa River water system (830304). The description is the same as for S1 Fig.
(TIF)

S8 Fig The regression coefficient function for the water system 830307. The function *β* ( *t* ) , with 95*%* confidence intervals for the Sagami River water system (830307). The description is the same as for S1 Fig.
(TIF)

S3 Table The *R* ^2^ values of the annual sum of flood damage. The *R*^2^ values are for each division into periods and each value of the number of days before each flood event. The *R*^2^ values were calculated for the entire study period (1993–2020) for all the river systems. ID denotes a pair of two periods for each division into periods. The red text highlights the optimal division into periods and number of days.
(TIF)

S9 Fig Annual variation of the total budget for river improvement in all of Japan. For the nation-wide river systems (1975–2023). The horizontal and vertical axes show the year and the budgetary amount, respectively. Created based on the statistical data from (MLIT. River Data Book; 2023 [cited 2024 Mar.]. https://www.mlit.go.jp/river/toukei_chousa/kasen_db/pdf/2024/2-4-4.pdf). Abbreviation: JPY, Japanese yen.
(TIF)

S10 Fig Annual variations of the budget for river improvement for each river system. For each river system under national government control (2014–2020). The horizontal and vertical axes show the year and the budgetary amount, respectively. The budgetary amount indicates per unit river basin area. Created based on the data from (MLIT. Budget for River Improvement Project; 2023 [cited 2024 Mar.]. https://www.mlit.go.jp/river/basic_info/yosan/gaiyou/yosan/index.html). The river systems corresponding to the codes in the legend are listed in S3 Table. Abbreviation: JPY, Japanese yen.
(TIF)
