# Correction: Historical precipitation and flood damage in Japan: Functional data analysis and evaluation of models

**DOI:** 10.1371/journal.pone.0321855

**Published:** 2025-04-01

**Authors:** Atsushi Wakai, Yasuaki Hijioka, Masayuki Yokozawa, Manabu Watanabe, Gen Sakurai

The corresponding author’s email address is incorrect. The correct email address is: sakurai.gen270@naro.go.jp.

## References

[1] Wakai A, Hijioka Y, Yokozawa M, Watanabe M, Sakurai G. Historical precipitation and flood damage in Japan: functional data analysis and evaluation of models. PLoS One. 2025;20(2): e0318335. doi: 10.1371/journal.pone.0318335 39999184 PMC11856516

