# Supplementary figures and images for: Historical precipitation and flood damage in Japan: functional data analysis and evaluation of models
Source: PLoS One. 2025 Feb 25;20(2):e0318335. doi: 10.1371/journal.pone.0318335 (PMC11856516; doi:10.1371/journal.pone.0318335)

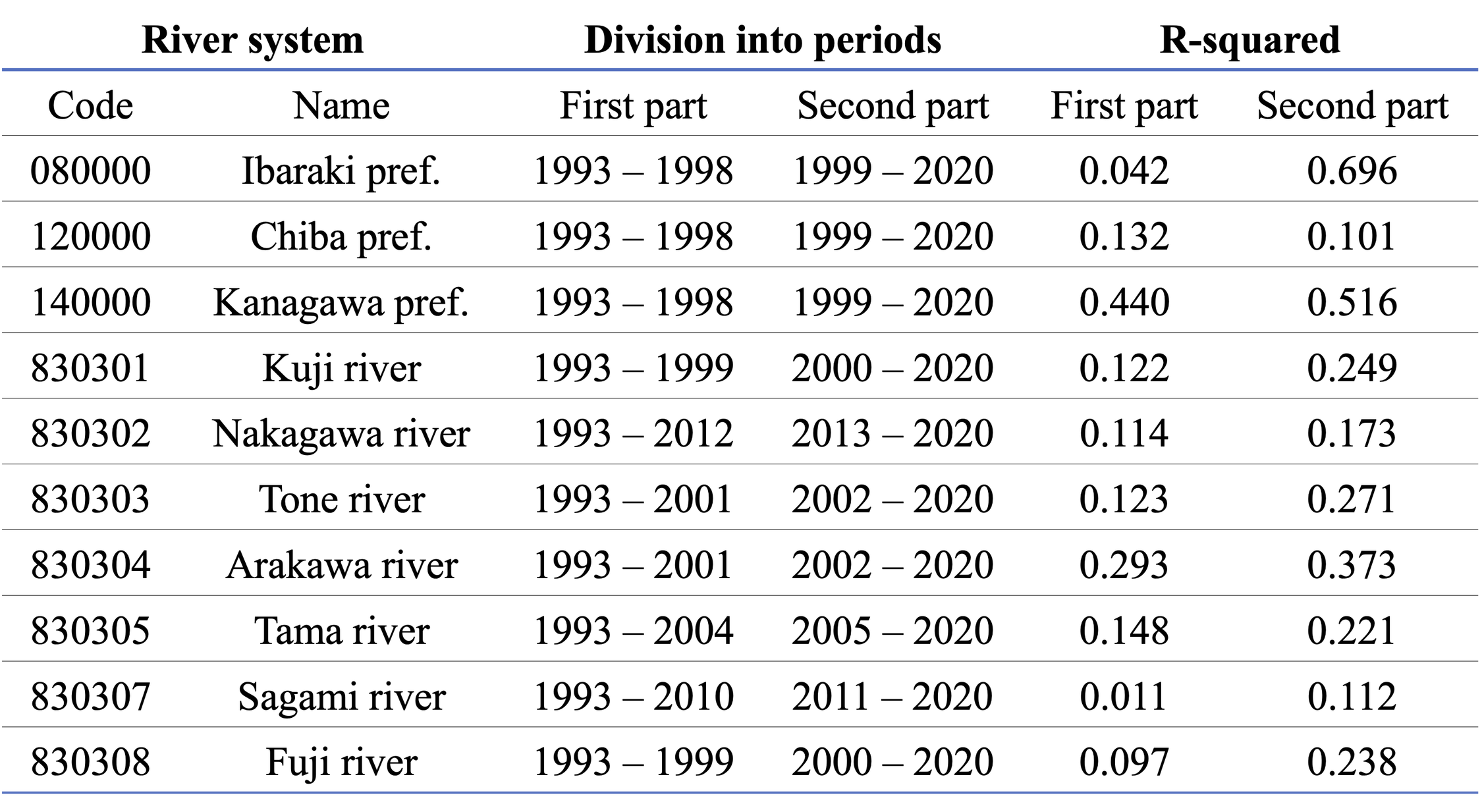

Supplement: S1 Table — The example that a response variable was the damage per gross regional product (GRP) in each river basin. Abbreviation: Pref., Prefecture. The historical GRP data at the prefectural level were obtained from a database provided by the Cabinet Office (Prefectural economic statistics; 2024. https://www.esri.cao.go.jp/jp/sna/sonota/kenmin/kenmin_top.html). The data contain information such as the gross product for each prefecture in each year. The GRP in each river basin was calculated by multiplying the GRP for the target region by the ratio of the population within the river basin to the population for the target region. The GRP was adjusted to the value of the Japanese yen in the year 2020. (TIF) [file pone.0318335.s001.tif]

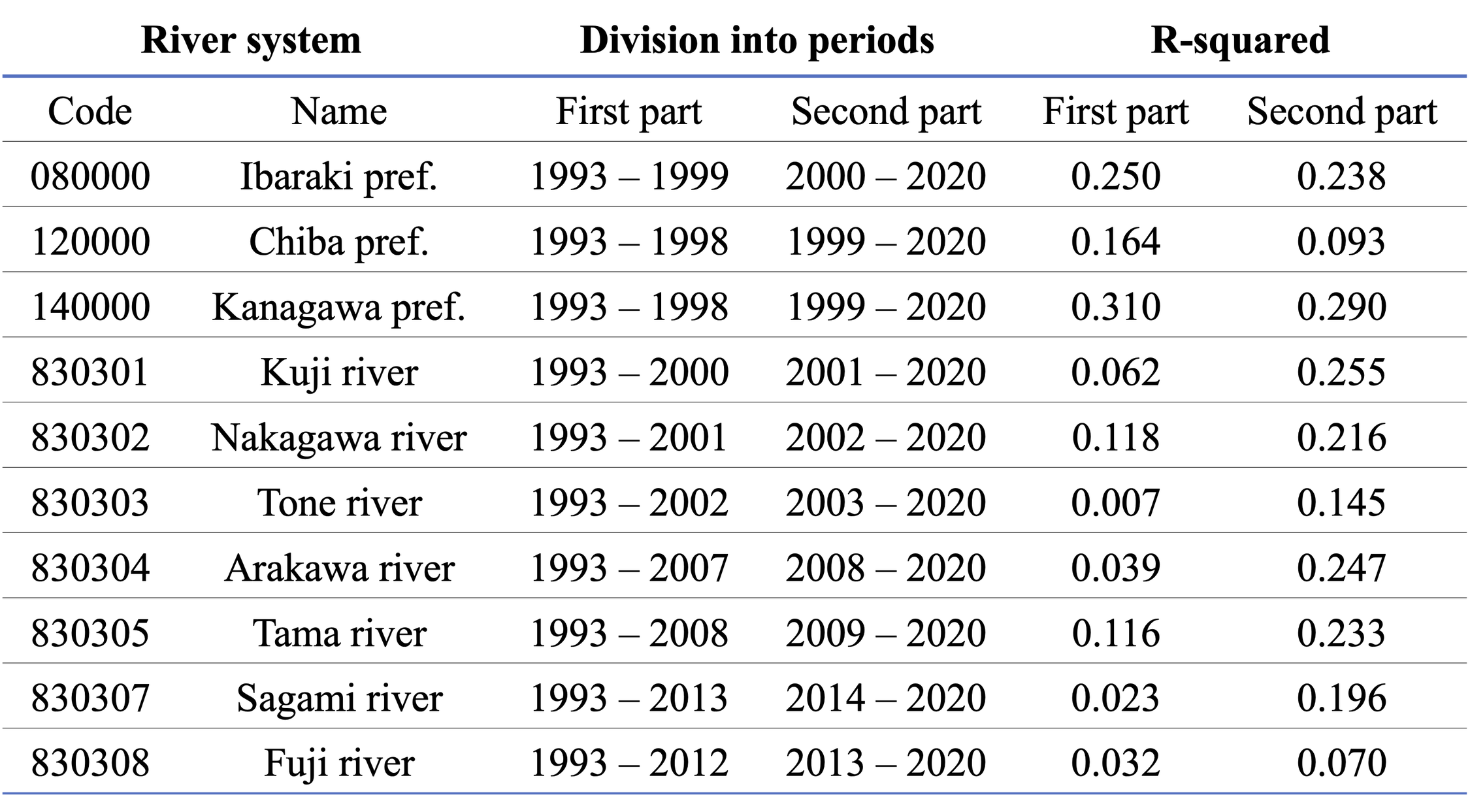

Supplement: S2 Table — The example that a response variable was the damage per unit area in each river basin. Abbreviation: Pref., Prefecture. (TIF) [file pone.0318335.s002.tif]

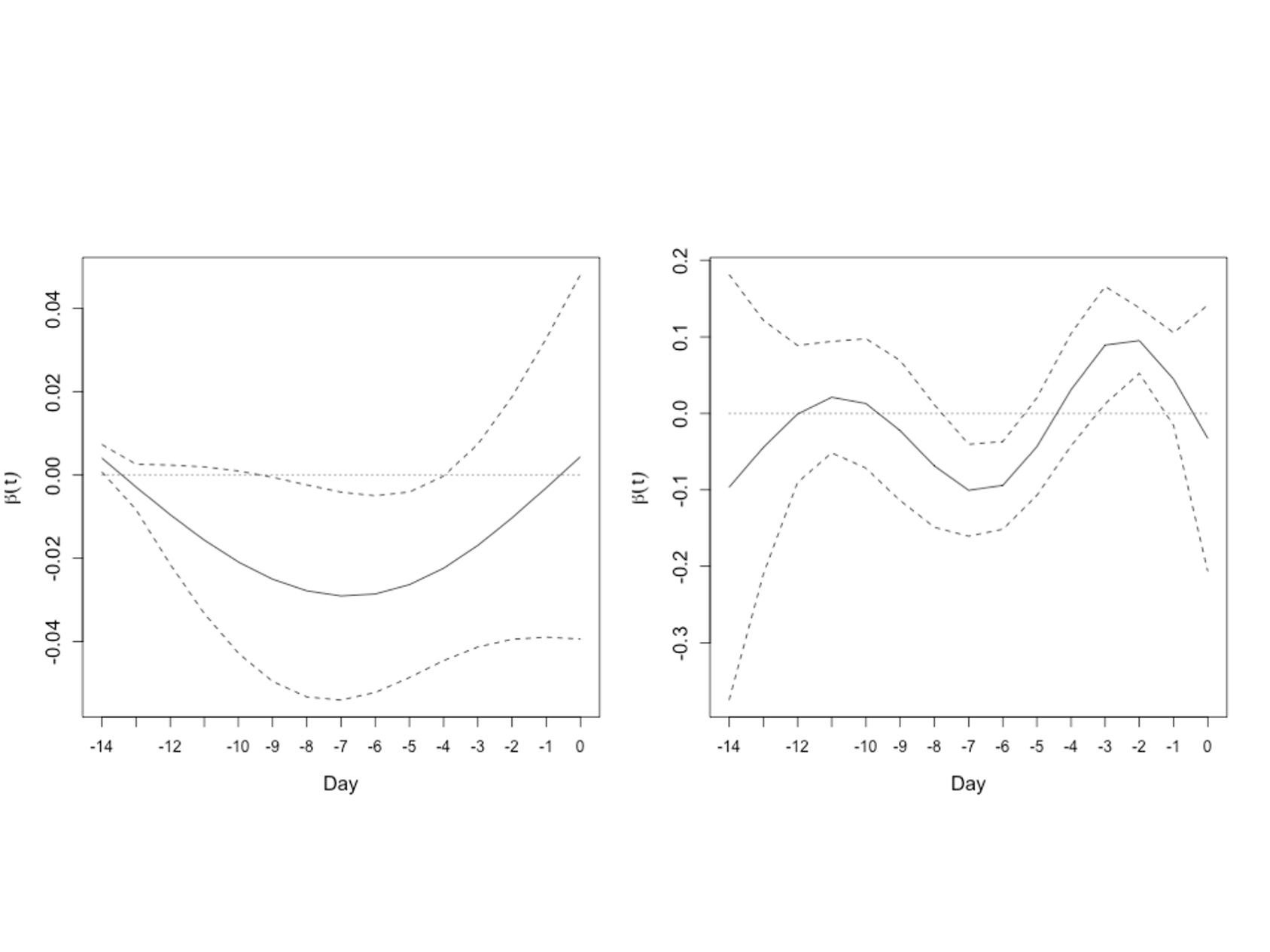

Supplement: S1 Fig — The function β ( t ) , with 95% confidence intervals for the Ibaraki Prefecture water system (080000) for the optimal division into periods and the optimal number of days before each flood event. The left and right panels show 1993–1999 and 2000–2020, respectively. The horizontal and vertical axes represent the number of days before each flood event and the values of β ( t ) , respectively. Day 0 represents the day when each flood event occurred. Solid lines represent the average regression coefficient function; dashed lines denote 95% confidence intervals. (TIF) [file pone.0318335.s003.tif]

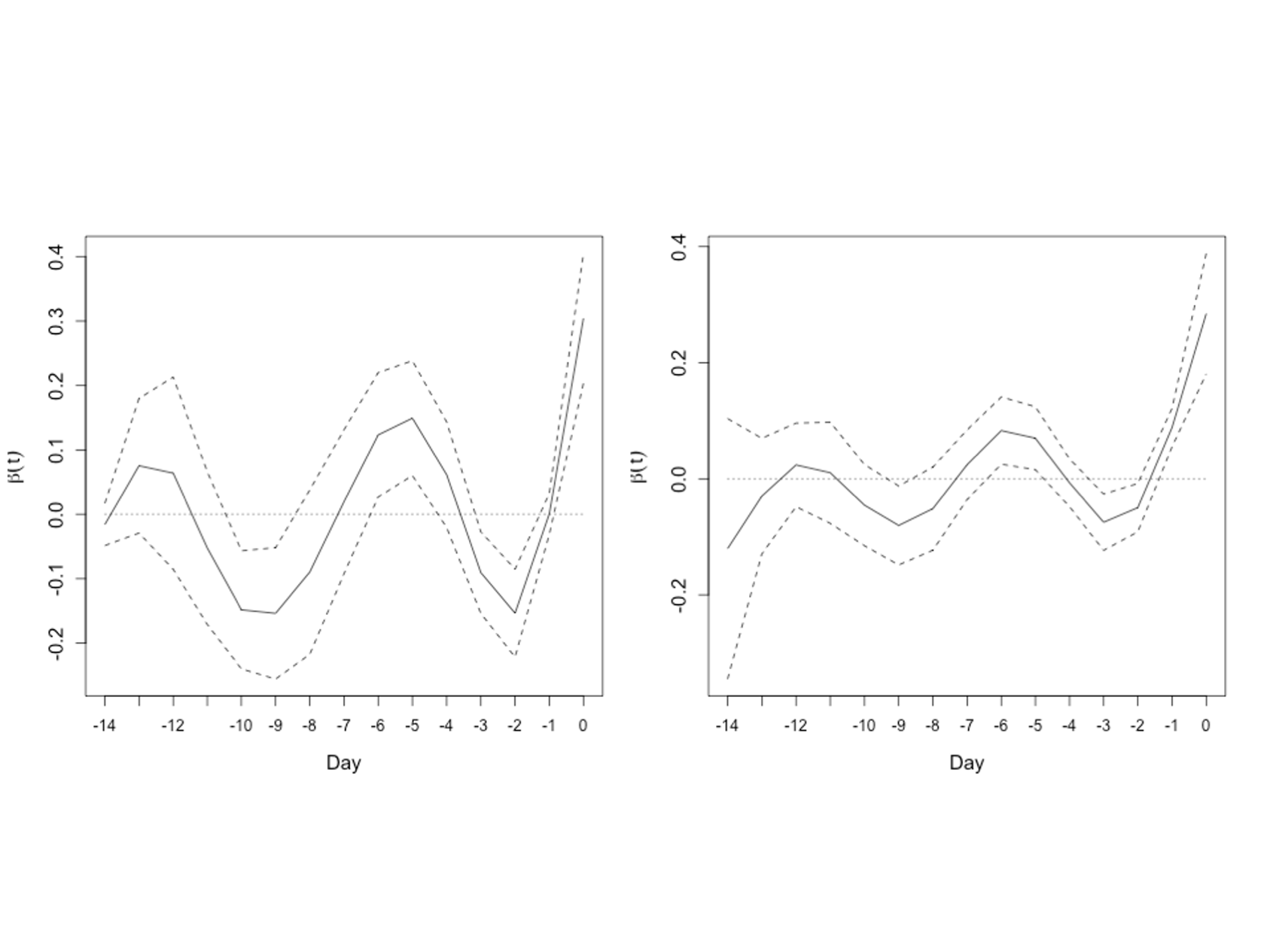

Supplement: S2 Fig — The function β ( t ) , with 95% confidence intervals for the Chiba Prefecture water system (120000). The description is the same as for S1 Fig. (TIF) [file pone.0318335.s004.tif]

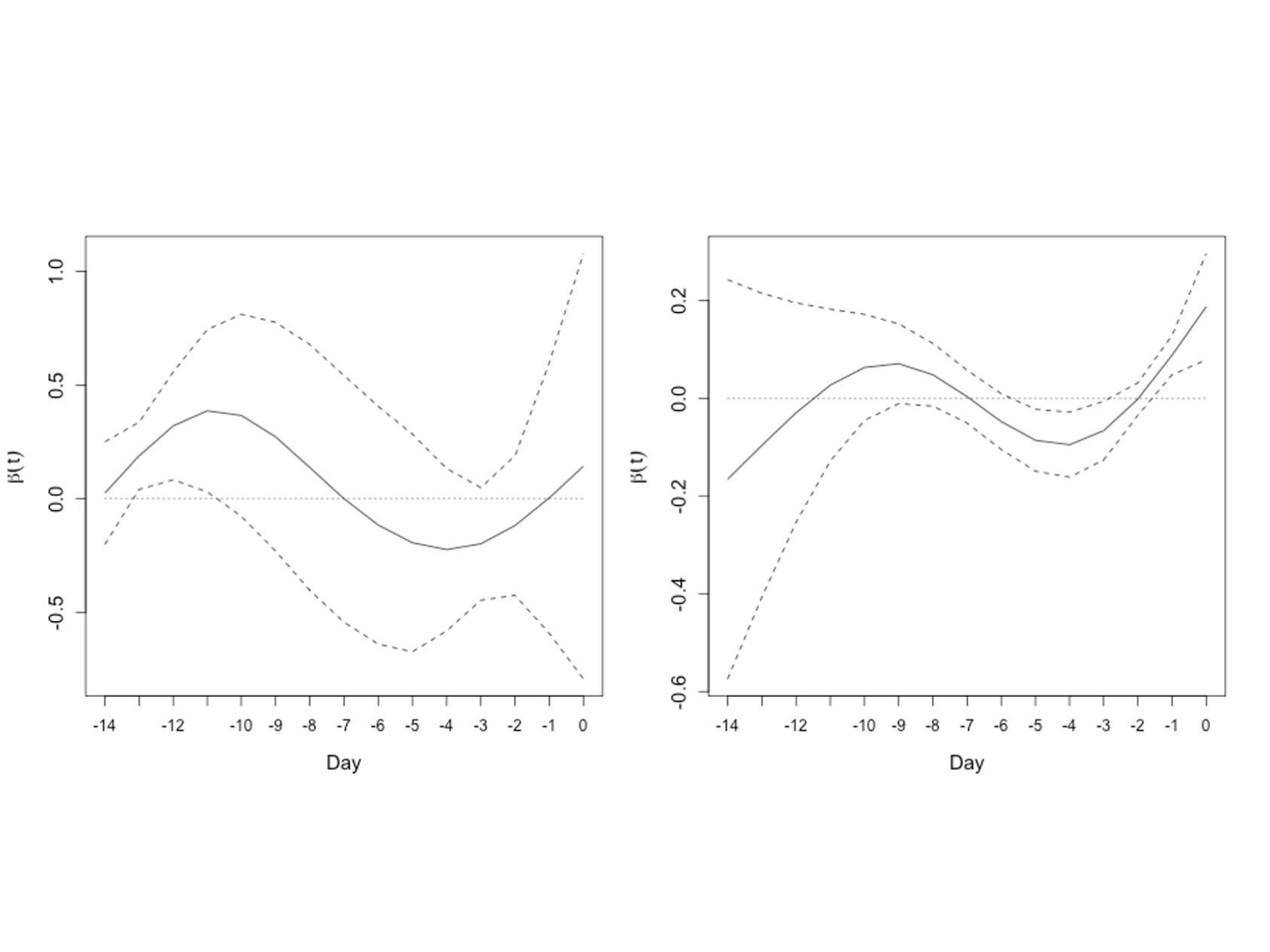

Supplement: S3 Fig — The function β ( t ) , with 95% confidence intervals for the Kanagawa Prefecture water system (140000). The description is the same as for S1 Fig. (TIF) [file pone.0318335.s005.tif]

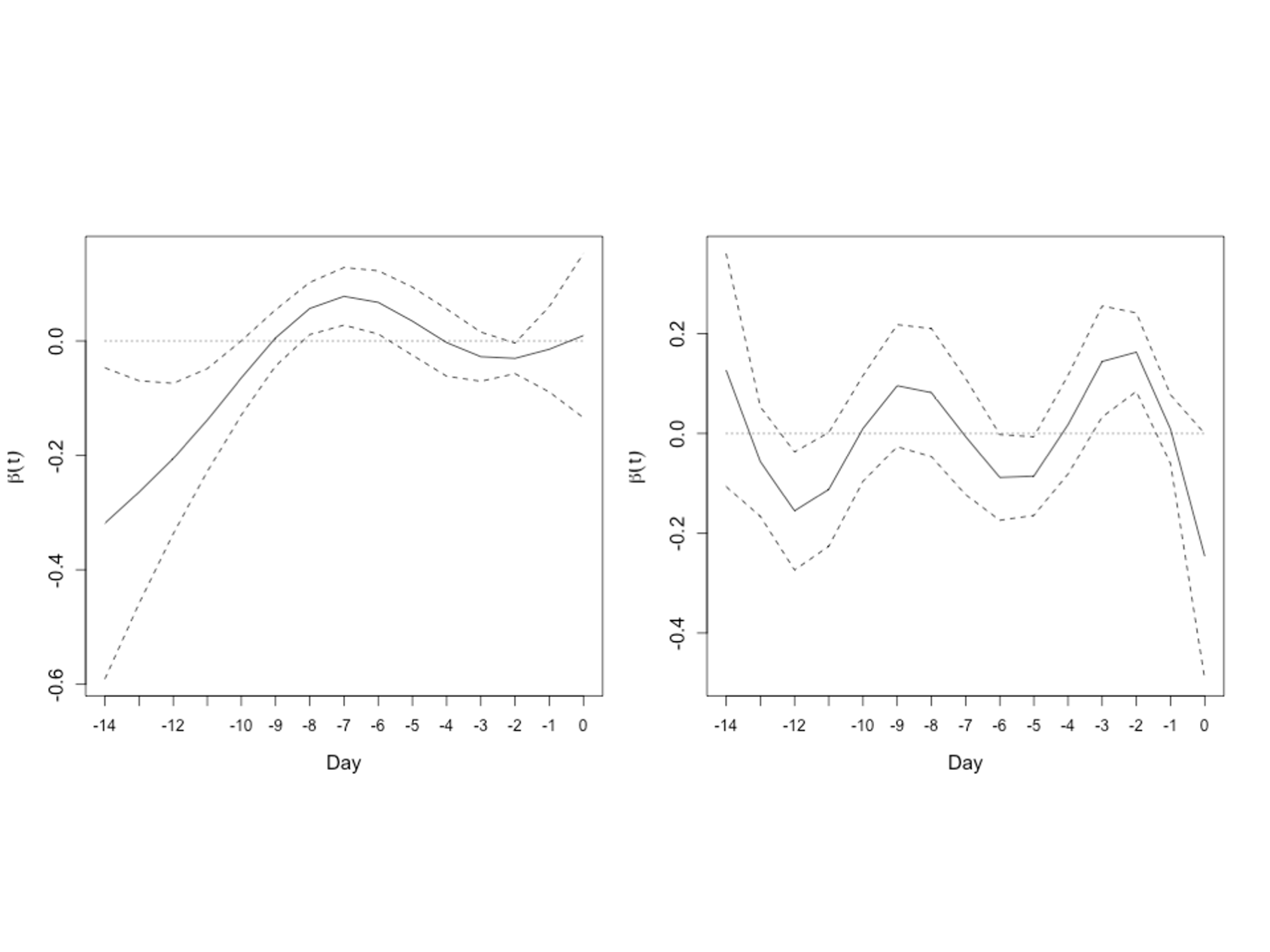

Supplement: S4 Fig — The function β ( t ) , with 95% confidence intervals for the Kuji River water system (830301). The description is the same as for S1 Fig. (TIF) [file pone.0318335.s006.tif]

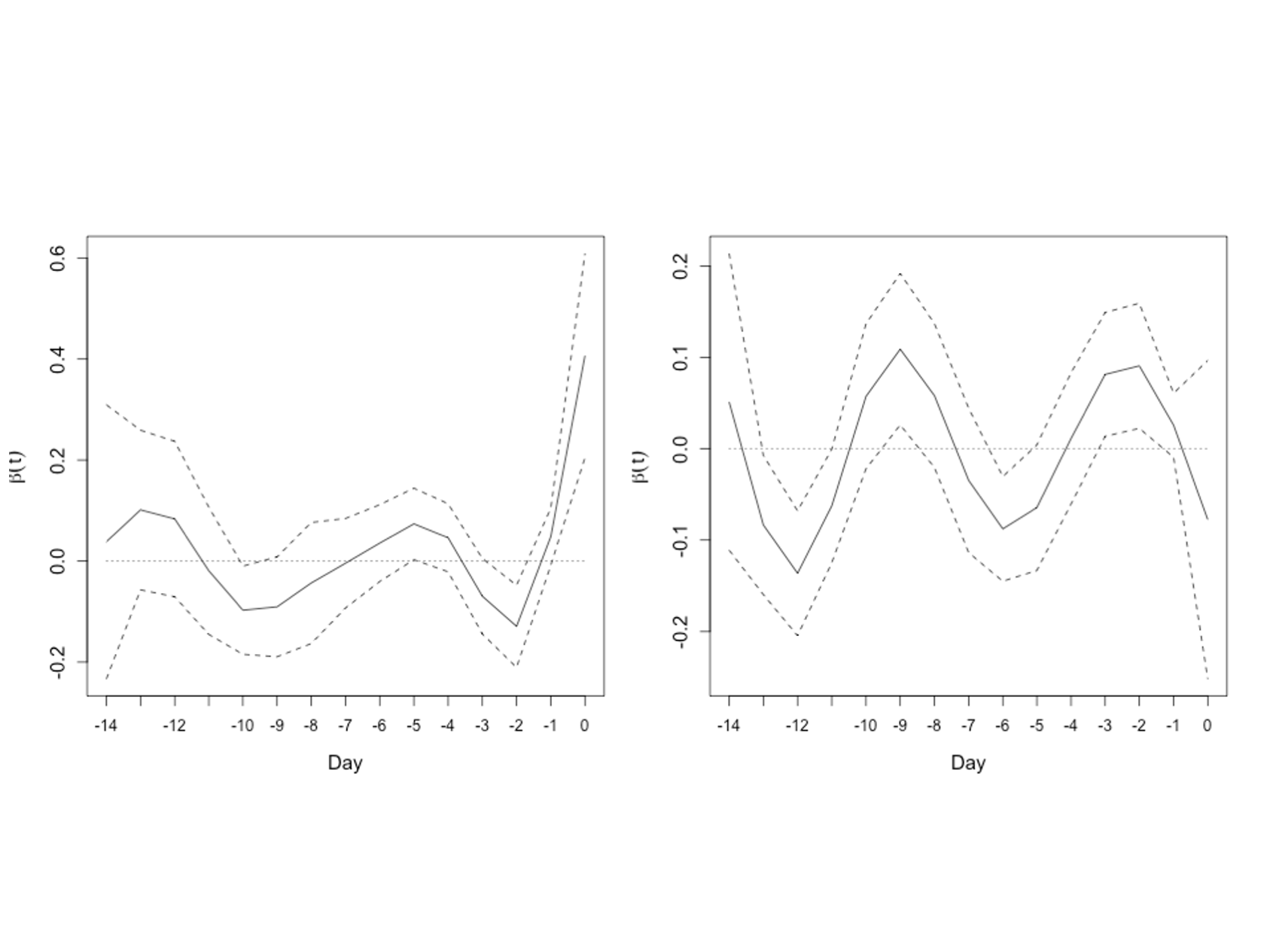

Supplement: S5 Fig — The function β ( t ) , with 95% confidence intervals for the Naka River water system (830302). The description is the same as for S1 Fig. (TIF) [file pone.0318335.s007.tif]

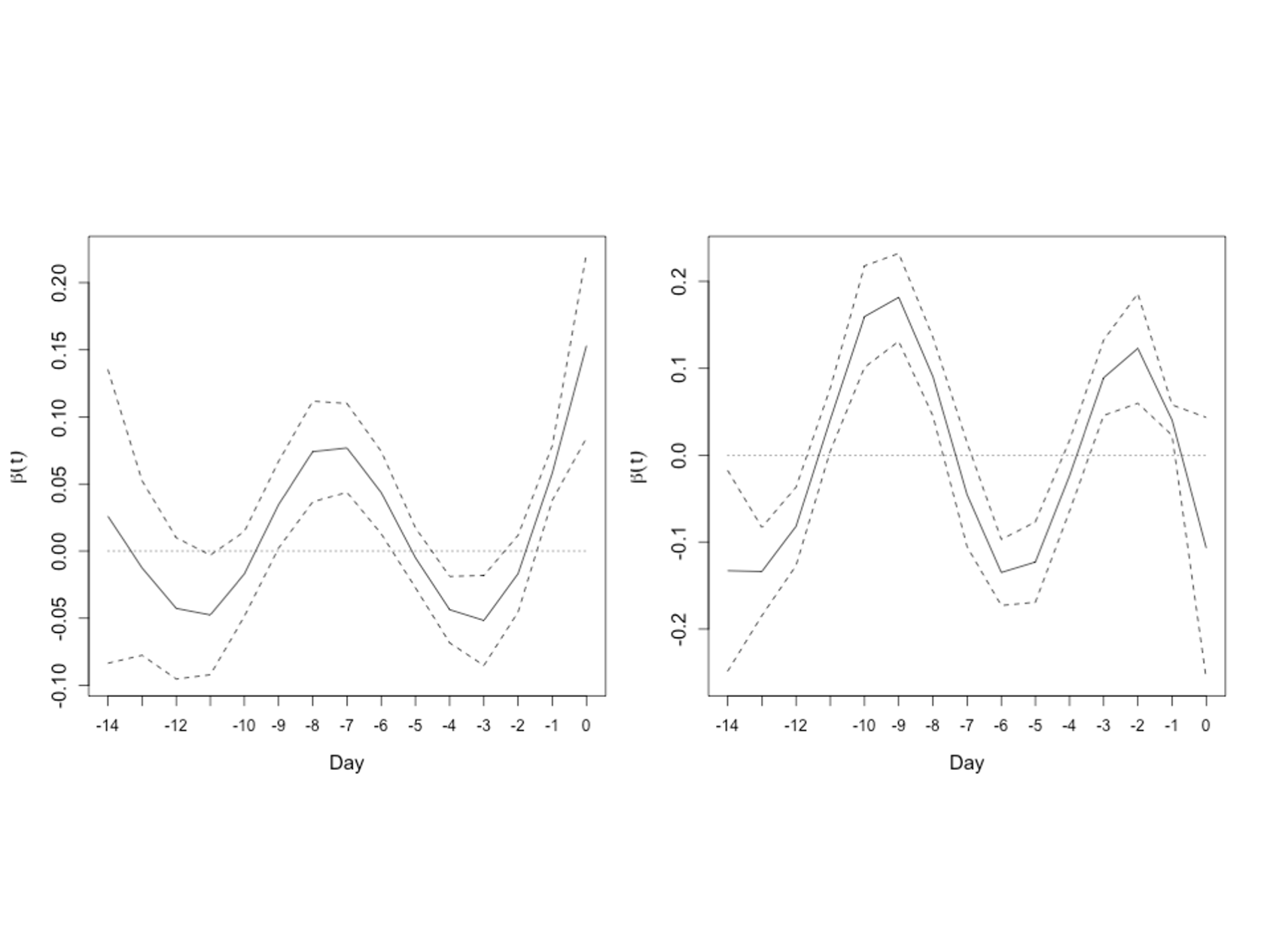

Supplement: S6 Fig — The function β ( t ) , with 95% confidence intervals for the Tone River water system (830303). The description is the same as for S1 Fig. (TIF) [file pone.0318335.s008.tif]

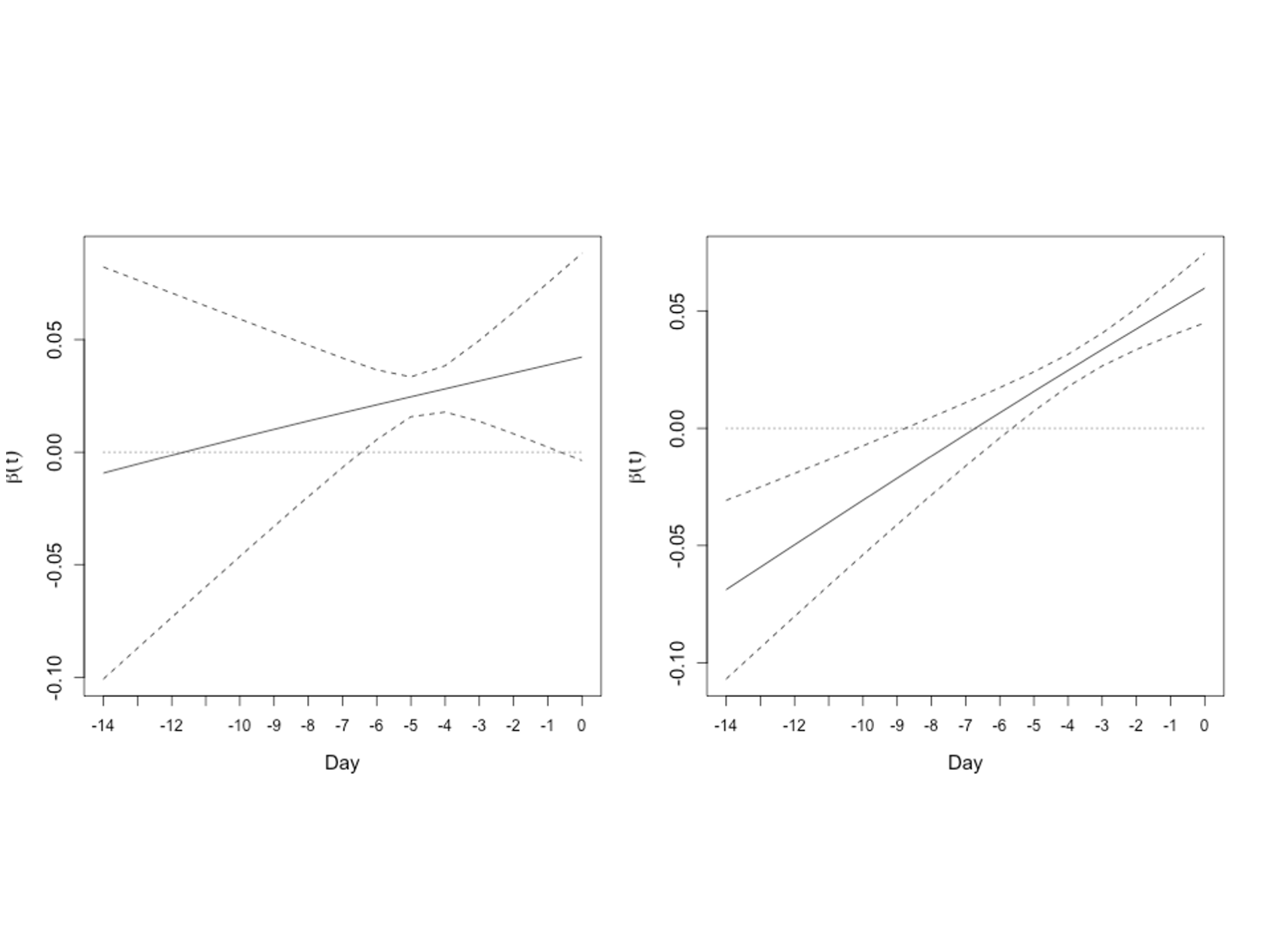

Supplement: S7 Fig — The function β ( t ) , with 95% confidence intervals for the Arakawa River water system (830304). The description is the same as for S1 Fig. (TIF) [file pone.0318335.s009.tif]

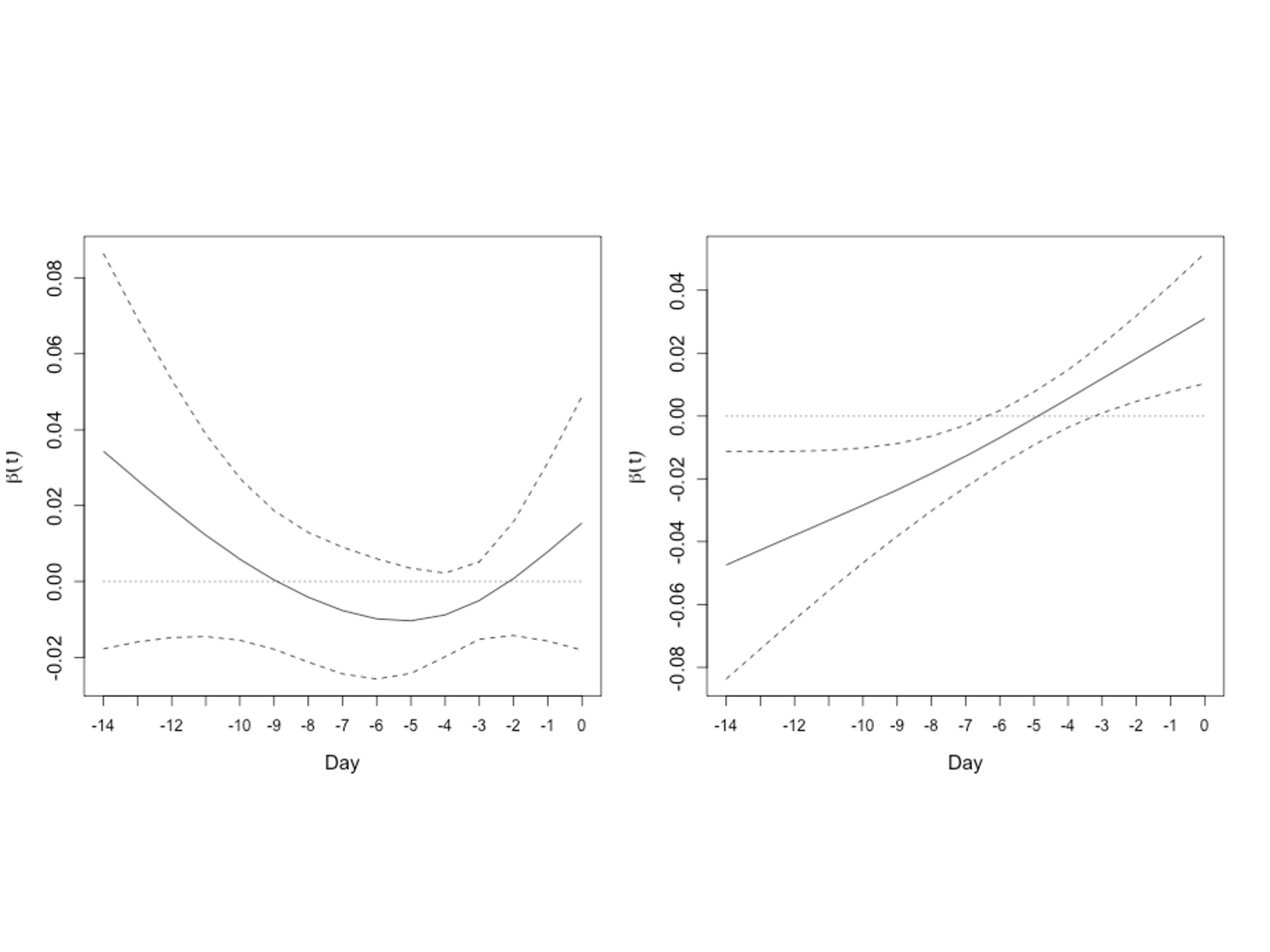

Supplement: S8 Fig — The function β ( t ) , with 95% confidence intervals for the Sagami River water system (830307). The description is the same as for S1 Fig. (TIF) [file pone.0318335.s010.tif]

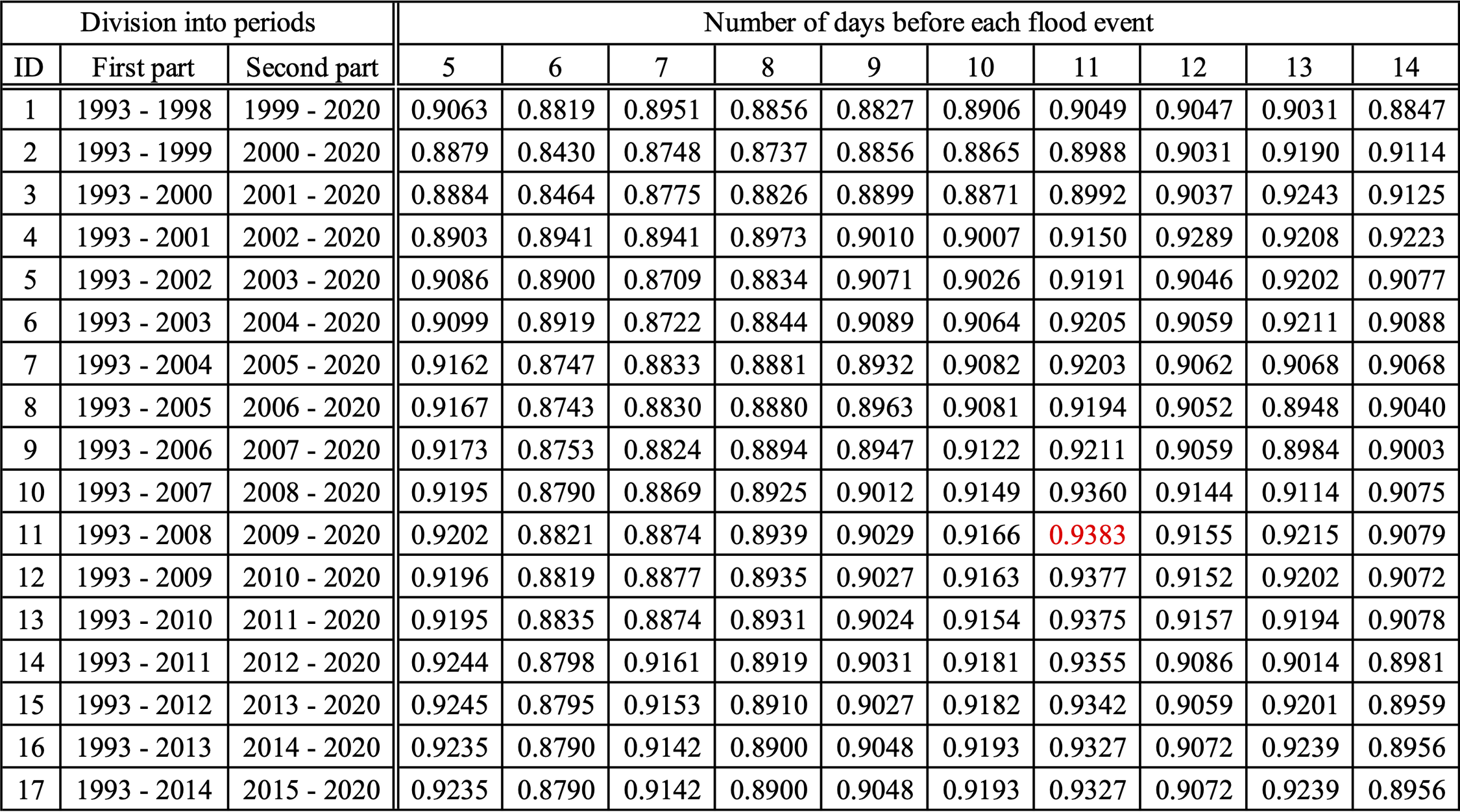

Supplement: S3 Table — The R2 values are for each division into periods and each value of the number of days before each flood event. The R2 values were calculated for the entire study period (1993–2020) for all the river systems. ID denotes a pair of two periods for each division into periods. The red text highlights the optimal division into periods and number of days. (TIF) [file pone.0318335.s011.tif]

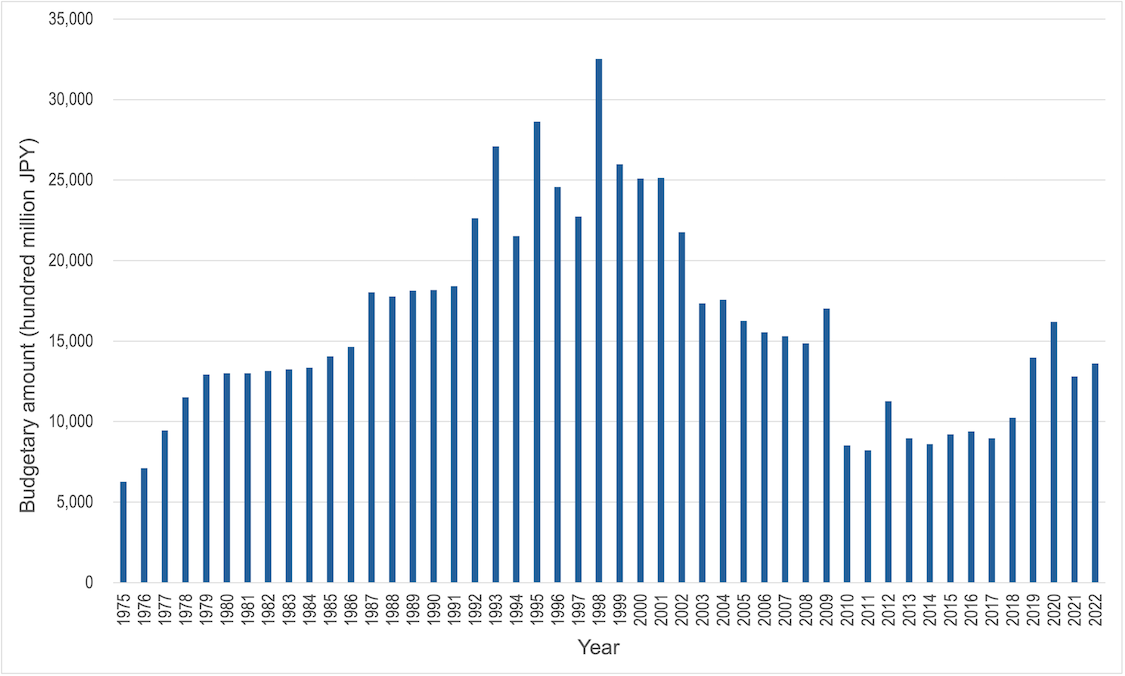

Supplement: S9 Fig — For the nation-wide river systems (1975–2023). The horizontal and vertical axes show the year and the budgetary amount, respectively. Created based on the statistical data from (MLIT. River Data Book; 2023 [cited 2024 Mar.]. https://www.mlit.go.jp/river/toukei_chousa/kasen_db/pdf/2024/2-4-4.pdf). Abbreviation: JPY, Japanese yen. (TIF) [file pone.0318335.s012.tif]

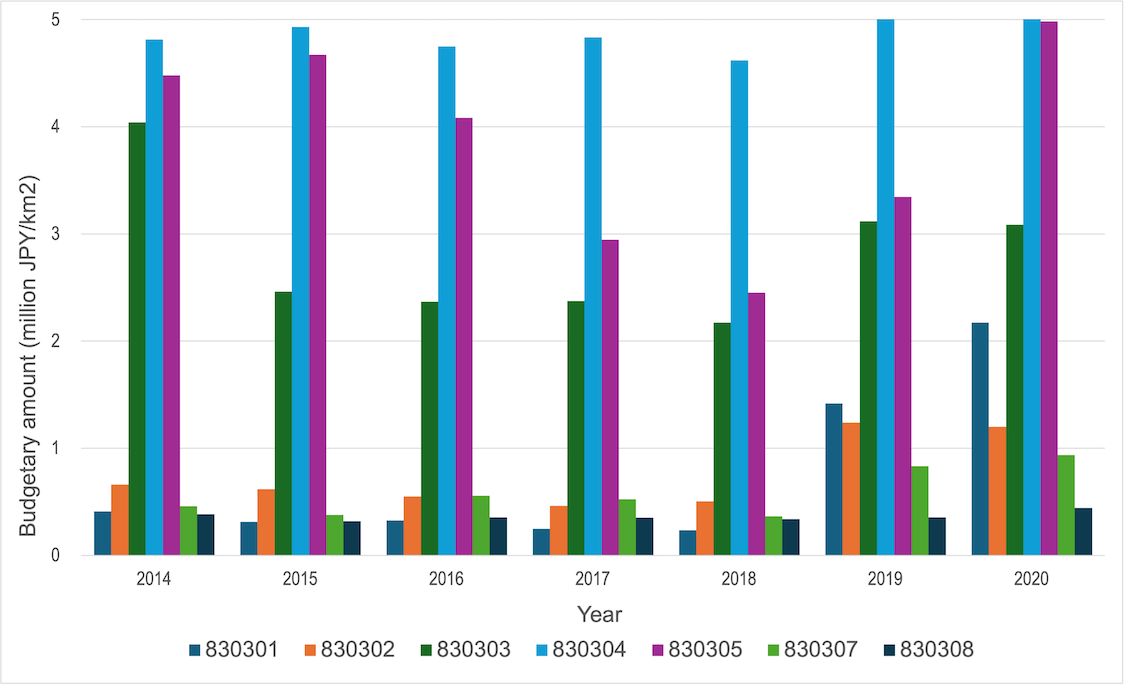

Supplement: S10 Fig — For each river system under national government control (2014–2020). The horizontal and vertical axes show the year and the budgetary amount, respectively. The budgetary amount indicates per unit river basin area. Created based on the data from (MLIT. Budget for River Improvement Project; 2023 [cited 2024 Mar.]. https://www.mlit.go.jp/river/basic_info/yosan/gaiyou/yosan/index.html). The river systems corresponding to the codes in the legend are listed in S3 Table. Abbreviation: JPY, Japanese yen. (TIF) [file pone.0318335.s013.tif]
